# Alternative venous access sites for dual-lumen extracorporeal membrane oxygenation cannulation

**DOI:** 10.1093/icvts/ivae060

**Published:** 2024-04-11

**Authors:** Armin-Kai Schoeberl, Dawid Staudacher, Mitsuaki Kawashima, Courtney Fischer, Marcelo Cypel, Nina Buchtele, Thomas Staudinger, Clemens Aigner, Konrad Hoetzenecker, Thomas Schweiger

**Affiliations:** Department of Thoracic Surgery, Medical University of Vienna, Vienna, Austria; Department of Cardiothoracic and Vascular Surgery, Kepler University Hospital, Medical Faculty Johannes Kepler University Linz, Linz, Austria; Department of Intensive Care Medicine, University of Freiburg, Freiburg, Germany; Department of Thoracic Surgery, University of Toronto, Toronto, ON, Canada; Department of Thoracic Surgery, University of Toronto, Toronto, ON, Canada; Department of Thoracic Surgery, University of Toronto, Toronto, ON, Canada; Department of Medicine I, Intensive Care Unit, Medical University of Vienna, Vienna, Austria; Department of Medicine I, Intensive Care Unit, Medical University of Vienna, Vienna, Austria; Department of Thoracic Surgery, Medical University of Vienna, Vienna, Austria; Department of Thoracic Surgery, Medical University of Vienna, Vienna, Austria; Department of Thoracic Surgery, Medical University of Vienna, Vienna, Austria

**Keywords:** Dual-lumen cannula, Dual-lumen catheter, Extracorporeal membrane oxygenation, Venous access, Lung transplantation, Respiratory failure

## Abstract

**OBJECTIVES:**

Dual-lumen cannulas for veno-venous (VV) extracorporeal membrane oxygenation (ECMO) support are typically inserted in the right internal jugular vein (RIJV); however, some scenarios can make this venous route inaccessible. This multicentre case series aims to evaluate if single-site cannulation using an alternative venous access is safe and feasible in patients with an inaccessible RIJV.

**METHODS:**

We performed a multi-institutional retrospective analysis including high-volume ECMO centres with substantial experience in dual-lumen cannulation (DLC) (defined as >10 DLC per year). Three centres [Freiburg (Germany), Toronto (Canada) and Vienna (Austria)] agreed to share their data, including baseline characteristics, technical ECMO and cannulation data as well as complications related to ECMO cannulation and outcome.

**RESULTS:**

A total of 20 patients received alternative DLC for respiratory failure. Cannula insertion sites included the left internal jugular vein (*n* = 5), the right (*n* = 7) or left (*n* = 3) subclavian vein and the right (*n* = 4) or left (*n* = 1) femoral vein. The median cannula size was 26 (19–28) French. The median initial target ECMO flow was 2.9 (1.8–3.1) l/min and corresponded with used cannula size and estimated cardiac output. No procedural complications were reported during cannulation and median ECMO runtime was 15 (9–22) days. Ten patients were successfully bridged to lung transplantation (*n* = 5) or lung recovery (*n* = 5). Ten patients died during or after ECMO support.

**CONCLUSIONS:**

Alternative venous access sites for single-site dual-lumen catheters are a safe and feasible option to provide veno-venous ECMO support to patients with inaccessible RIJV.

**Figure:**
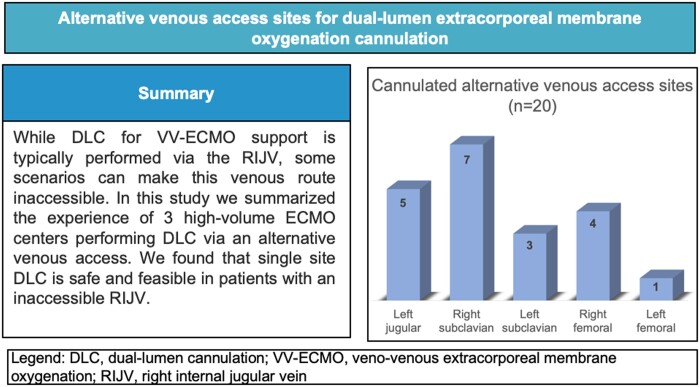

## INTRODUCTION

Veno-venous (VV) extracorporeal membrane oxygenation (ECMO) support is an established treatment for patients suffering from severe respiratory failure [1]. While two-site cannulation, including a drainage cannula and a separate return cannula, remains the standard ECMO configuration, single-site dual-lumen cannulas are commonly used by many ECMO centres [2–6]. A major hurdle of dual-lumen cannulation (DLC) is the sometimes challenging insertion which requires visualization with fluoroscopy or echocardiography. The intended insertion site of DLC is the right internal jugular vein (RIJV) due to the relatively straight line from the superior vena cava to the right atrium and inferior vena cava [7, 8]. After puncturing the RIJV, the cannula must be advanced from the superior vena cava to the inferior vena cava without deviation to the right ventricle. Accidental introduction to the ventricle can lead to fatal cardiac injuries [9–11]. Despite the rather difficult insertion, single-site dual-lumen catheters confer several advantages as compared to conventional VV-ECMO configuration, such as improved patient mobilization and reduced recirculation [2, 3, 10]. Increased mobilization and early weaning from mechanical ventilation can prevent further debilitation which is particularly important for patients requiring prolonged ECMO support as a bridge to recovery or lung transplantation [12–15].

Since an accessible and patent RIJV is considered a prerequisite for DLC by most centres, it is usually not performed in patients with thrombosis or stenosis of the RIJV. Some centres, however, have reported successful DLC insertion using alternative venous access sites such as the left internal jugular vein (LIJV) or subclavian vein. However, currently available literature on alternative DLC is limited to 2 case reports and a small single-centre case series [16–18]. Thus, data on feasibility and safety that include a relevant number of patients are currently missing. This study aims to summarize a multi-institutional experience with alternative venous access sites for dual-lumen catheters including 3 high-volume ECMO centres. Furthermore, we aimed to explore the indications, techniques and complications in patients with this rare type of ECMO configuration.

## PATIENTS AND METHODS

### Study design

We performed a retrospective, multicentre analysis of adult patients receiving a dual-lumen catheter for VV-ECMO support via an alternative venous access between January 2011 and April 2022. Alternative venous access was defined as LIJV, left or right subclavian vein and left or right femoral vein. In a first step, 16 high-volume ECMO centres with an annual use of 10 or more dual-lumen cannulas were approached. Three centres [Freiburg (Germany), Toronto (Canada) and Vienna (Austria)] agreed to share their data including gender, age, weight, body mass index (BMI), underlying diagnosis, type of respiratory failure, treatment goal, reason for alternative cannulation duration and outcome. Additionally, technical cannulation data, ECMO flow parameters, respiratory parameters and data regarding complications related to ECMO cannulation including the need for cannula repositioning, vascular injury during insertion, bleeding at cannulation site, thrombosis at cannula, thromboembolism, air embolism, relevant flow limitation and need for change of ECMO configuration were collected. This study was approved by the ethics committee of the Medical University of Vienna (EK-Nr: 1877/2021). Informed consent was waived by the local ethics committee due to the retrospective nature of the study.

### Cannulation technique

**Figure 1:**
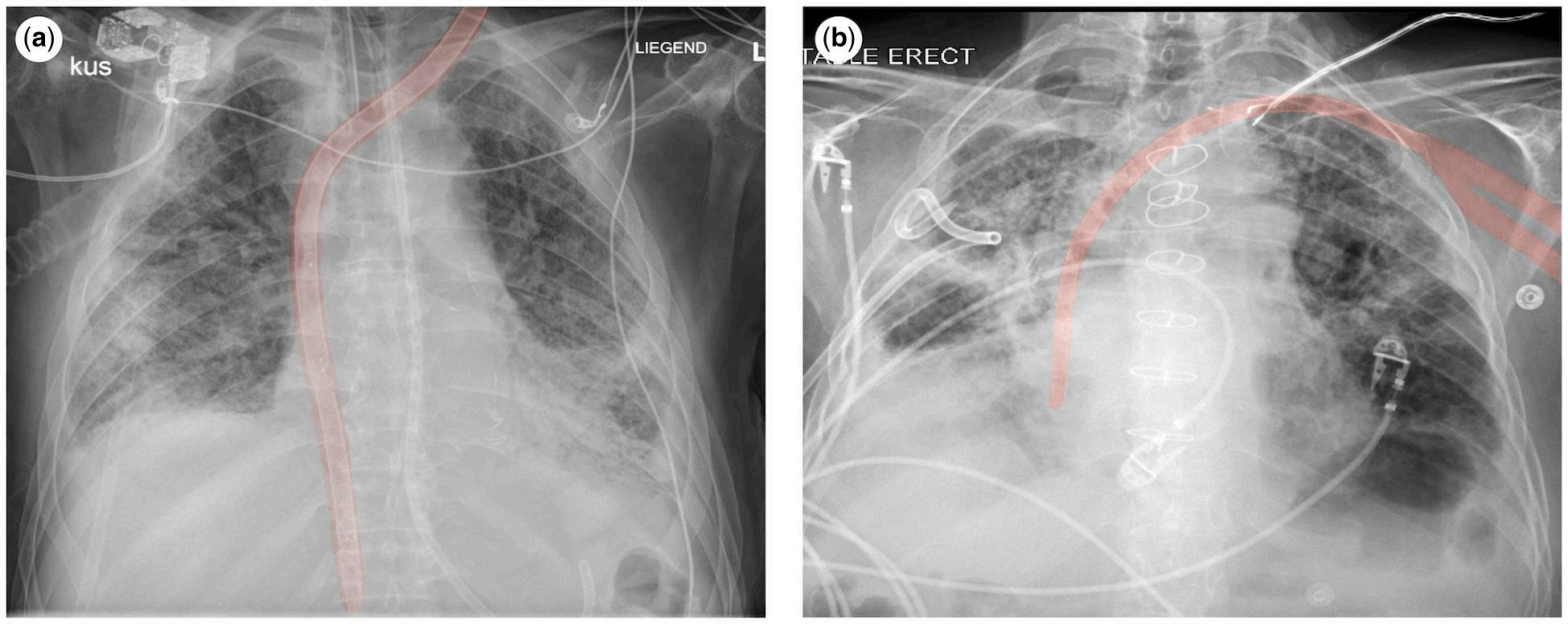
Chest X-ray of a 28 FR Crescent cannula inserted via the LIJV in an 58-year-old patient with hypercapnic and hypoxemic respiratory failure and thrombosis of the RIJV (**a**) and chest X-ray of an 19 FR Avalon cannula inserted via the left subclavian vein in an 61-year-old patient with hypercapnic respiratory failure (**b**). FR: French; LIJV: left internal jugular vein; RIJV: right internal jugular vein.

Cannulation was uniformly performed in Seldinger technique. After universal prepping and draping the area for venous access, the vein was punctured and a guidewire was introduced. In cases of cannulation of the jugular or subclavian vein, the guidewire should eventually traverse through the superior vena cava, the right atrium and finally the inferior vena cava. To ensure adequate placement of the guidewire and avoid potentially fatal complications by deviation of the cannula, imaging was performed in the form of transoesophageal echocardiography, fluoroscopy or sequential bedside X-rays depending on the ECMO team. An appropriately sized skin incision was performed and either serial dilators with increasing size or the unisize dilatator included in the cannulation set were introduced. Next, without retrieving the guidewire, the dual-lumen cannula (Avalon, Getinge AB, Sweden or Crescent, Metronic, USA) was advanced until the desired position was reached. After confirming the correct position of the cannula by assessing the depth and the position of the radiopaque markers the cannula was connected to the ECMO circuit and secured with sutures (Fig. 1). In case of femoral cannulation, either a 19- or 20-French (FR) cannula was placed as proximal in the inferior vena cava as possible, which in our experience required the introduction of the entire length of the cannula and resulted in the cannula reaching the hepatic venous confluence.

### Statistical analysis

Descriptive statistics were used for baseline characteristics. Categorical variables are reported as percentages and counts; continuous variables are reported as median and interquartile range. The software SPSS Statistics Version 27.0.1.0 (IBM) was used for statistical analyses.

## RESULTS

### Study population

A total of 20 patients were included in the final analysis. Nine cases were reported by the ECMO team of the Toronto General Hospital (Canada), 5 cases by the Medical University of Freiburg (Germany) and 6 cases by the Medical University of Vienna (Austria). Ten patients were male and 10 patients were female with a median age of 55 (38–63) years at the time of cannulation. Reasons for VV-ECMO support included acute respiratory distress syndrome (ARDS) secondary to viral or bacterial pneumonia (*n* = 8), rejection after lung transplantation (*n* = 5), primary graft dysfunction (*n* = 2), exacerbation of pulmonary sarcoidosis (*n* = 1), exacerbation of interstitial lung disease (*n* = 1), ARDS due to aspiration (*n* = 1), ARDS after blunt tracheal injury (*n* = 1) and respiratory failure after lung volume reduction surgery (*n* = 1). Nine patients suffered from hypercapnic respiratory failure, 5 patients from hypoxemic respiratory failure and 6 patients had combined hypercapnic and hypoxemic respiratory failure. Demographic and clinical characteristics are summarized in Table 1.

**Table 1:** Patients’ characteristics.

Total cannulations (*n* = 20)		
	*n*	%
Age (years)^a^	55 (38–63)	
Body mass index^a^	24.5 (20.4–29.1)	
Sex		
Male	10	50
Female	10	50
Indication		
ARDS	10	50
Exacerbation of sarcoidosis or ILD	2	10
Rejection after LTX	5	25
Primary graft dysfunction	2	10
Respiratory failure after LVRS	1	5
Cannulation setting		
Intensive care unit	10	50
Operating room	7	35
Angiography suite	3	15
Respiratory failure		
Hypoxemic	5	25
Hypercapnic	9	45
Combined	6	30
Ventilation		
Intubated	13	65
Non-intubated/awake	7	35
Cannula type and size		
Avalon—19 FR	6	30
Avalon—20 FR	3	15
Avalon—27 FR	3	15
Avalon—28 FR	1	5
Avalon—31 FR	2	10
Crescent—24 FR	1	5
Crescent—28 FR	4	20
Insertion site		
Left jugular	5	25
Left subclavian	3	15
Right subclavian	7	35
Left femoral	1	5
Right femoral	4	20
Guiding and imaging		
Sequential X-ray	4	20
Fluoroscopy	10	50
Transoesophageal echocardiography	6	25
Duration of ECMO support (days)	15 (9–22)	

a Median (25th, 75th percentile).

ARDS: acute respiratory distress syndrome; ECMO: extra corporal membrane oxygenation; FR: French; ILD, interstitial lung disease; LTX: lung transplantation; LVRS: lung volume reduction surgery.

### Alternative extracorporeal membrane oxygenation cannulation and parameters

Reasons for alternative cannulation were thrombosis or stenosis of RIJV (*n* = 6), occupation of RIJV by a central line (*n* = 4), inaccessible RIJV due to trauma and secondary subcutaneous emphysema (*n* = 1), assumed increased patient comfort with subclavian cannulation (*n* = 3) and change of ECMO configuration with occupation of RIJV by a primary return cannula (*n* = 4). In 2 cases, the reason for the alternative cannulation was not specified. Patients were cannulated either in the intensive care unit (*n* = 10), the operating room (*n* = 7) or the angiography suite (*n* = 3). Venous access was achieved via the LIJV (*n* = 5), the right (*n* = 7) or left (*n* = 3) subclavian vein and the right (*n* = 4) or left (*n* = 1) femoral vein. Fourteen patients were cannulated with an Avalon cannula and 6 patients received a Crescent cannula. The median initial target ECMO flow was 2.9 l/min (1.8–3.1) and corresponded with individual cannula size and cardiac output. The median cannula size was 26 (19–31) FR. The maximum median ECMO flow was 3.0 l/min (2.0–3.9) and the median ECMO runtime was 15 (9–22) days. Cannula sizes and achieved flow rates are available in Table 2.

**Table 2:** Cannula size and extra corporal membrane oxygenation flow parameters.

Parameters	19 FR	20 FR	24 FR	27 FR	28 FR	31 FR
*N*	6 (30%)	3 (15%)	1 (5%)	3 (15%)	5 (25%)	2 (10%)
BMI	26.5 (23.2–28.3)	20.6 (15.2–30.4)	29.8	25.9 (21.5–30.8)	21.9 (19.8–27.4)	22.8 (21.6–23.9)
Initial flow (l/min)	1.8 (1.6–1.9)	1.8 (1.5–2.9)	3.0	3.1 (2.7–3.5)	3.0 (2.9–3.3)	4.8 (3.9–5.6)
Max. flow (l/min)	2 (1.9–2.0)	1.8 (1.5–3.8)	4.0	3.3 (2.9–3.6)	4.0 (3.4–4.6)	5.1 (4.1–6.0)

BMI: body mass index; FR: French.

### Complications and outcome

No procedural complications were reported. During ECMO support, 1 patient developed bleeding at the cannulation site that eventually required blood transfusion and was managed by adapting the heparin dosage. One patient suffered from recurring ‘suck down’ episodes of the ECMO cannula, which was managed with continuous volume administration. The cannula was first repositioned and ultimately changed to a femoral-jugular configuration. Unfortunately, this did not reduce the frequency of ‘suck down’ episodes and the patient had to further be managed with additional volume administration. While 13 patients were sedated and mechanically ventilated via an endotracheal tube, a total of 7 patients were awake on ECMO support. One out of these 7 patients received respiratory support via a tracheostomy, 6 patients did not require any mechanical ventilation. A total of 10 patients (50%) survived to decannulation. Five patients were successfully bridged to lung transplantation and 5 patients were successfully bridged to recovery. Ten patients died during or after ECMO support. Reasons for death were failure to recover and withdrawal of therapy (*n* = 4), multi-organ failure (*n* = 1), patient will (*n* = 1), anoxic brain injury (*n* = 2), unclear cerebral oedema (*n* = 1) and haemorrhagic shock after a liver biopsy (*n* = 1) (Table 3).

**Table 3:** Complications and outcome.

Patients (n=20)	*N*	%
Complications		
Bleeding at cannulation site	1	5
Change of ECMO configuration	1	5
Repositioning	1	5
Outcome		
Bridge to LTX	5	25
Bridge to recovery	5	25
Failure to recover	4	20
Death for other reasons	6	30

ECMO: extra corporal membrane oxygenation; LTX: lung transplantation

## DISCUSSION

**Figure 2:**
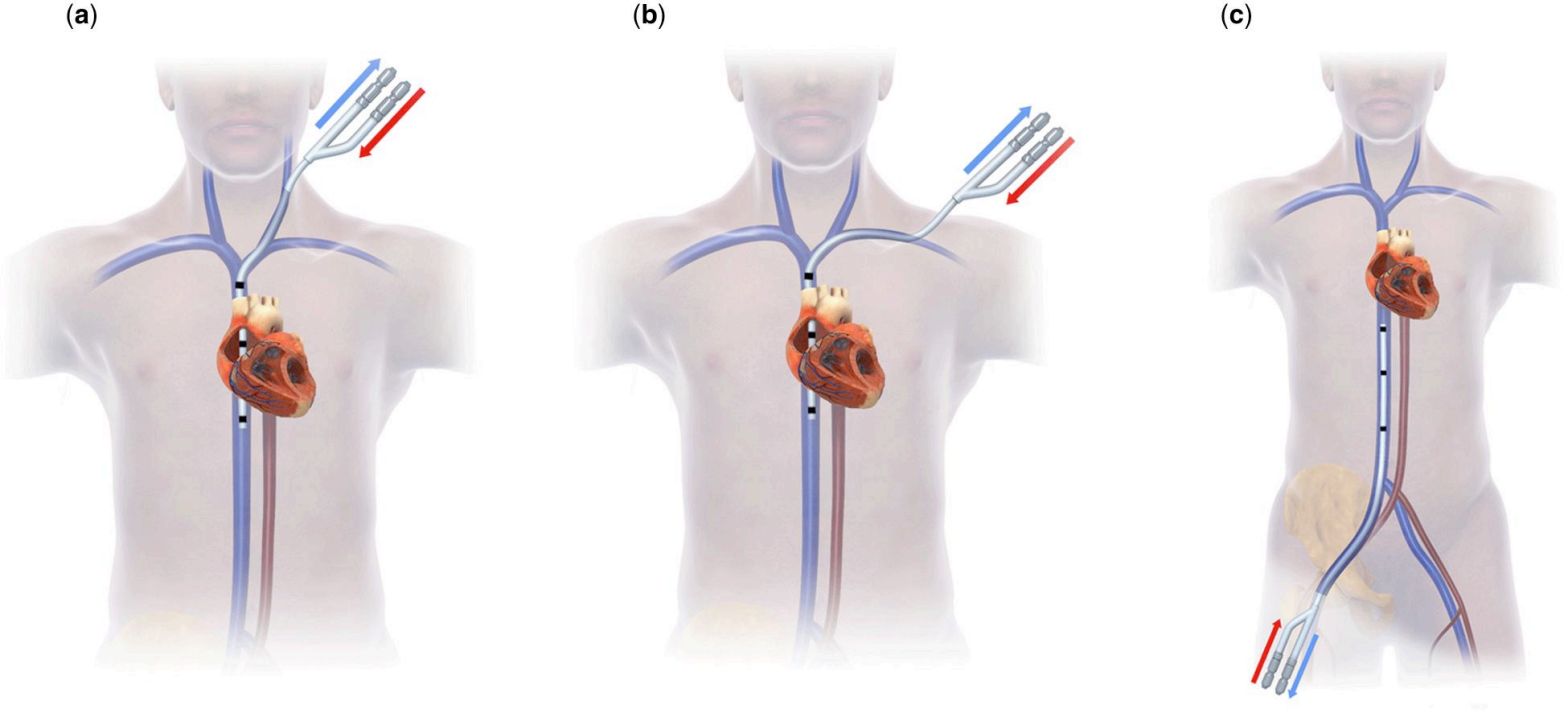
Display of alternative cannulation strategies for dual-lumen cannulation; access via the left internal jugular vein (**a**), the left subclavian vein (**b**) and the right femoral vein (**c**).

In this multicentre study, we demonstrated the feasibility and safety of alternative cannulation sites for DLC VV-ECMO support in the so far largest patient cohort (Table 4). Patients with hypercapnic and hypoxemic respiratory failure were successfully treated with VV-ECMO support with a dual-lumen catheter inserted into either the LIJV, subclavian vein or femoral vein (Fig. 2). Our findings show that adequate ECMO flow can be achieved with dual-lumen catheters cannulated via an alternative venous access.

**Table 4:** Literature review of alternative cannulations sites for dual-lumen extra corporal membrane oxygenation cannulation.

Author	Year	Patients	Cannula type	Cannula size	Venous access	Imaging	Reason for alternative cannulation	Complications
Shafii and McCurry [16]	2012	3	Avalon	31 FR	Left subclavian vein	Flouroscopy	Patient size	1 case of thrombosis in the left internal jugular vein
Abrams *et al.* [17]	2012	4	Avalon	23, 27 FR	LIJV	Flouroscopy and TEE	Haematoma, stenosis, or thrombosis of RIJV	None
Chan *et al.* [18]	2020	1	Crescent	28 FR	Left subclavian vein	Flouroscopy and TEE	Change from dual site for increased ambulation	None
Schoeberl *et al.*	2024	20	Avalon and Crescent	19–31 FR	LIJV, left and right subclavian vein, left and right femoral vein	Flouroscopy, sequential X-ray and TEE	Multiple reasons, see publication for further information	1 case of cannulation site bleeding, 1 case of repositioning and change of ECMO configuration

ECMO: extracorporeal membrane oxygenation; FR: French; LIJV: left internal jugular vein; RIJV: right internal jugular vein; TEE: transoesophageal echocardiography.

Shafii and McCurry [16] first described the cannulation of the 31-cm long Avalon cannula through the subclavian vein as a solution to offer adequately sized dual-lumen catheters to smaller patients. Additionally, they reported increased patient mobility and comfort with subclavian vein cannulation. Abrams *et al.* [17] meanwhile demonstrated successful DLC in 4 patients with inaccessible RIJV using a 23 or 27 FR Avalon cannula inserted through the LIJV. In 2020, Chan *et al.* demonstrated successful transition from dual-site VV-ECMO support, to a single-site dual-lumen cannula inserted into the left subclavian vein. They concluded that the left subclavian vein is a viable option to change ECMO configuration to maximize patient mobility and comfort when prolonged ECMO course is predicted [18]. While these reports indicate that dual-lumen catheters are still efficient when used at an alternative insertion site, larger case studies are missing to date.

The fear of potential fatal complications such as vascular or right-sided heart injury may result in hesitations performing alternative DLC [9–11]. However, so far none of these fatal complications was described in patients with alternative cannulation sites. One reason could be an underreporting of such fatal complications. On the other hand, these exceptional cannulations are primarily carried out at well-experienced centres with established cannulation protocols. In cases with complete occlusion of the RIJV, DLC via the LIJV or the subclavian vein poses a theoretical risk of superior inflow congestion. Nevertheless, since most inserted cannulas do not fully occlude the superior central venous system and multiple venous collaterals are able to divert the blood flow, this complication was not observed in patients cannulated via an alternative venous access [19]. In this series, transoesophageal echocardiography, fluoroscopy and sequential bedside X-rays were used as imaging tools while performing cannulation. All 3 methods seemed to be viable techniques to achieve adequate imaging and guiding dependent on the ECMO team [20]. The low complication rate in the literature as well as in this study cohort provides valuable evidence that despite the unfavourable anatomic conditions, VV-ECMO support with a dual-lumen catheter can be considered in patients with inaccessible RIJV.

It is important to mention that sole CO_2_ removal using small bore dual-lumen catheters is a well-established and commonly applied extra corporal life support configuration for extracorporeal CO_2_ elimination (ECCO2R). These catheters can be inserted at any venous access site and are capable of reducing CO_2_ in in patients with hypercapnic lung failure [21]. In the present cohort however, more than half of the patients required complete lung replacement for severe hypoxic lung failure. This requires considerable higher flow rates and therefore bigger cannula sizes. The ECMO flow rates in this patient cohort were adapted to meet the patients individual requirements and resulted in a median ECMO flow rate of 2.9 l/min. Changing of ECMO configuration due to flow limitations in the form of repeated ‘suck down’ episodes was only necessary in 1 case. Dual-lumen catheters inserted into a femoral vein and therefore not reaching up the right atrium, appeared to also be able to provide sufficient ECMO performance in patients with moderate hypoxaemia. It remains speculative, whether the improved oxygenation occurs secondary after sufficient decarboxylation and thus improves pulmonary perfusion. Furthermore, a sufficient amount of oxygenated blood might still reach the right atrium despite the drainage port located proximally to the infusion port.

### Limitations

Several limitations of this study have to be discussed. First, the retrospective design might confer a selection bias and limit the quality of the retrieved data. Moreover, the number of included patients is still low. Despite merging data from 3 high-volume ECMO centres, the resulting patient cohort lacks statistical power to allow an in-depth analysis and comparison with a control group. Even within the included patients, management and treatment strategies might have varied between the contributing institutions. Considering the rare occurrence of these extraordinary situations, evidence from prospective studies cannot be expected in the future. In summary, single-site dual-lumen catheters cannulated using an alternative venous access are a safe and effective option to provide sufficient VV-ECMO support in selected patients with inaccessible RIJV.

## Data Availability

Data is available upon reasonable request from the corresponding author.

## Notes

Presented at the 37th EACTS Annual Meeting in Vienna, Austria from 4-7 October 2023.

## ABBREVIATIONS

ARDS: Acute respiratory distress syndrome
BMI: Body mass index
DLC: Dual-lumen cannulation
ECMO: Extracorporeal membrane oxygenation
FR: French
LIJV: Left internal jugular vein
RIJV: Right internal jugular vein
VV: Veno-venous
